# Tenth Annual Meeting of the American Medical Association

**Published:** 1857-06

**Authors:** 


ART. III.— *Tenth Annual Meeting of the American Medical Association.*

This body convened in the Representative Hall of the State Capitol, at Nashville, Tenn., on Tuesday, May 5th, at 11 o'clock, A. M.

The association was called to order by Dr. Pitcher, of Detroit.

The chairman then announced that the first business in order was the report of the Committee of Arrangements.

Dr. Winston, on behalf of that committee, took the stand, and in a few brief and pertinent remarks, welcomed to the city the delegates present.

The chairman of the Committee of Arrangements then called the roll, and the following delegates answered to their names:

*Connecticut* — Charles Hooker.

*New Hampshire*—Adoniram Smalley.

*New York*—James R. Wood, D. M. Reese, Geo. N. Burwell, and Alden March.

*Pennsylvania*—Robey Dunglison, B. F. Schenck, Casper Wistar, and P. Cassidy.

*Georgia*—Henry F. Campbell, C. R. Walton, N. P. Powers, A. H. Means, Joseph P. Logan, M. H. Oliver, Thomas Powell, J. Gordon Howard, R. D. Arnold, Geo. P. Padleford, and Pike Brown.

*Alabama*—G. M. Merriweather, W. P. Reese, A. F. Alexander, S. W. Clanton, W. H. Thornton, P. C. Winn, T. Stith Malone, W. J. Bass, G. D. Norris, J. F. Sowell, and J. W. Morris.

*Tennessee*—Frank A. Ramsey, James Rodger, R. O. Currey, B. B. Lanoir, J. L. C. Johnston, John M Boyd, George R. Grant, T. A. Atchison, S. S. Mayfield, J. D. Kelley, T. L. Maddin, J. D. Winston, J. E. Manlove, G. A. J. Mayfield, Richard Owen, W. P. Jones, G. F. Jones, J. S. Burford, Jno. John P. Ford, Robert C. Foster, John H. Callendar, John H. Morton, A. H. Buchanan, J. W. Hoyte, N. C. Perkins, John B. Lindsley, C. K. Winston, Paul F. Eve, (permanent member), W. P. Moore, Milo Smith, Wallace Estill, B. W. Avent, H. H. Clayton, H. M. Whittaker, H. B. Manlove, T. M. Woodson, A. Ewing, Robert Martin, W. K. Bowling, P. S. Woodward, R. F. Evans, Thomas Lipscomb, M. Ransom, J. A. Long, John M. Watson, W. D. Haggard, John S. Park, D. B. Cliff, T. G. Kennedy, T. R. Jennings, Ira Conwell, W. H. Childress, W. A. Cheatham, J. F. Towns, J. M. Brannock, B. C. Willson, P. W. Davis.

*Louisiana*—S. O. Scruggs, Robert A. New, Cornelius Beard, and E. D. Fenner.

*Kentucky*—Samuel Arman, R. W. Gaines, J. B. Flint, J. W. Singleton, R. J. Breckenridge, S. C. Porter, W. S. Chipley, S. M. Bemiss, L. G. Ray, W. A. Atchison, E. G. Davis, and L. E. Almon.

*Indiana*—W. H. Byford, W. W. Hitt, Isaac Mendenhall, T. Bullard, N. Johnson, D. W. Yandell.

*Illinois*—J. C. H. Hobbs, A. H. Luce, James M. Steel, E. K. Crothers, T. K. Edmiston, W. A. Hillis.

*Missouri*—S. Pollock, E. S. Fraser, John S. Moore, C. A. Pope.

*Michigan*—A. B. Palmer, L. G. Robinson, Zina Pitcher, Wm. Brodie, L. H. Cobb, M. Gunn, Lewis Davenport, P. Cline, M. D. Stebbins.

*Iowa*—Asa Horr. Wm. Watson, D. L. McGugin, J. C. Hughes.

*Ohio*—Henry F. Koehne, J. M. Mosgrove, B. S. Brown, D. Ferris.

*Wisconsin*—Hays McKinley, J. K. Bartlett.

*South Carolina*—E. R. Henderson, M. S. Moore, R. W. Gibbs, and R. Z. Bailey.

*Mississippi*—F. B. Sanford, J. S. Cain.

*New Jersey*—R. M. Cooper.

*Arkansas*—F. G. McGavock.

The chairman of the committee then presented the names of Dr. Felix Robinson, Dr. John Shelby and Dr. John Overton, of Davidson county, with the reqnest that they be elected permanent members of the association.

A motion was made to that effect, which prevailed, and the above-named fathers in the profession were invited to participate in the proceedings of the association.

The President here announced that the next business in order was the appointment of a committee of one from each State, to nominate suitable officers for the ensuing year.

A motion was made that the association take a recess, of fifteen minutes, for that purpose, which was carried.

Fifteen minutes having elapsed, the delegates from the different States returned, and presented the following committee:

Connecticut, Charles Hooker; New Hampshire, A. Smalley; Indiana, W. W. Hitt; Wisconsin, J. K. Bartlett; New York, James R. Woods; Michigan, A. B. Palmer; Missouri, J. O. Moore; Illinois, T. K. Edmiston; Kentucky, R. J. Breckenridge; Arkansas, F. G. McGavock; Ohio, B. S. Brown; South Carolina, R. W. Gibbes; Alabama, W. O. Reese; Mississippi, E. B. Shuford; New Jersey, R. M. Cooper; Louisiana, S. O. Scruggs; Pennsylvania, P. Cassidy; Georgia, Thomas S. Powell; Tennessee, J. B. Lindsley; Iowa, Asa Horr.

Dr. Pitcher, the President, then delivered his retiring address. We have not the space to even give a substance of his remarks; suffice it to say that it was able throughout abounding in eloquence, and containing many scientific facts which will be highly valuable to the profession.

A motion was made returning the thanks of the association for the able manner in which the retiring president had discharged his duty, and also the appointment of a committee to request his address for publication. Carried.

The Treasurer's report was read and received.

Dr. W. K. Bowling, from the Committee on Prize Essays, asked for further time to report. Granted.

Judge Catron being present, was, by motion, invited to occupy a seat by the President.

The President informed the association that Dr. F. Campbell Stewart, of N. Y., Dr. Alden March, of N. Y., Dr. Isidor Glück, of N. Y.. and Dr. Pancoast, of Penn., had baen appointed to represent this body in Foreign Scientific Bodies.

Under the head of Reports from Standing Committees, a letter was read from the chairman of the Committee on Medical Topography and Epidemics, Dr. J. C. Watson, of Maine, stating that he had not prepared a report, but would be prepared by the next meeting of the association.

J. F. Posey, from the same committee, from the State of Georgia, presented a report, which was received.

Communications from Peregrine Wroth, of Maryland, and W. F. Sutton, of Kentucky, on the same subject, were read and received.

The Nominating Committee returned, and presented the following officers for the ensuing year:

*President*—Dr. Paul F. Eve.

*Vice-presidents*—Dr. R. J. Breckenridge, Kentucky; Dr. D. M. Reese, New York; Dr. William H. Byford, Indiana; Dr. Henry F. Campbell, Georgia.

The appointment of Secretaries was deferred until the association determined the next place of meeting.

The association then adjourned until 9 o'clock A. M., May 6th.

---

Wednesday, May 6, 1857.

The minutes of yesterday were read and adopted.

The President and Vice-presidents elect were conducted to their seats. Prof. Eve, upon assuming his position, made a brief and pertinent address.

Dr. Winston read the names of additional delegates, who were admitted to seats.

Dr. Hooker apologized for the absence of his report upon Medical Topography, from the fact that he had supposed that he had three years to make it.

The Chair appointed Drs. Curry, Grant and Evans a Committee on Voluntary Contributions.

A communication was rea[illegible] from Dr. Mendenhall, Ohio, apologizing for no report on Medical Topography.

Dr. Winston read a communication from the agents of the Methodist Publishing House, inviting the association to inspect their establishment. Accepted.

Dr. Lindsley read an invitation from the Medical Faculty of Washington City, requesting the association to hold its meeting for 1858, in that city. Referred to the Committee on Nominations.

A resolution of thanks to ex-president Pitcher, was adopted.

Dr. Arnold, of Ga., presented an abstract of a Topographical report, from Dr. Posey, of Ga., which report was referred to the Committee on Publication.

Dr. Wood, of N. Y., presented reports from Dr. Luckley and Dr. W. Army, upon Topography of Washington Territory. Referred to the Committee on Voluntary Contributions.

The chair proceeded to call for reports from special committees, many of which were presented.

Dr. D. M. Reese, of New York, read an abstract of a report upon infant mortality. The report was referred to the Committee on Publication.

Dr. Hobbs, of Illinois, offered a resolution providing for the appointment of a Committee of Reference for all essays. After discussion it was indefinitely postponed.

A protest, signed by several members of the association, against the admission of delegates from Ogelthorpe Medical College of Savanah, was read, on the grounds of irregularity in its organization.

Dr. Benson, delegate from that institution, was allowed to address the association. He explained that sickness was the principal cause of vacancies in the chairs, and stated that the protest was signed by professors in, and graduates from, a college which was a competitor of Oglethorpe.

Dr. Benson said that one of the delegates was a graduate from Tennessee. Dr. B. was considerably excited, and demanded if the gentleman from Oglethorpe denied his assertion.

Order was called, and Dr. Benson explained that he had been informed as he had stated, and if wrong, he preferred to be corrected in a gentlemanly manner.

Dr. Buchanan, of Tennessee, regretted that unpleasant feelings had arisen, and hoped the matter would be dropped.

The protest was tabled, and subsequently referred to a committee of three, upon which no professor was to be appointed.

The venerable Dr. Felix Robertson appeared, and was conducted to a seat with the President.

The convention then proceeded with the call for reports, many of which were not presented, and further time was granted.

A report upon remedies to be adopted to correct the evils of coroner's inquests, was received by mail from Dr. A. J. Simmons, of the District of Columbia. Read and referred to the Committee on Publication.

The report was accompanied with a resolution providing for the appointment of a committee of three in each State or Territory, for the purpose of memorializing their respective Legislatures for the carrying out of the remedies proposed. Adopted.

Dr. F. Hinkle, of Penn., reported upon the use of cinchona in malarious diseases. Read and referred to the Committee on Publication.

Dr. H. F. Campbell, of Georgia, reported on nervous system in febrile diseases, continued.

A report was read and referred to the Committee on Voluntary Contributions, from Dr. George Luckley, U. S. A., on the Medical Topography of Washington Territory.

Also a report from Dr. James Cooper, on the Flora of Washington and Oregon Territories. Referred to same committee.

A communication was read and received from Dr. N. S. Davis, of Illinois, on the composition and properties of milk.

Dr. Singleton, of Kentucky, offered a resolution in reference to the death of Dr. F. J. Grafton, of Mississippi.

Dr. Reynolds, a member of the association, was expelled for certifying for a quack medicine.

The following preamble and resolution in reference to the decease of our late esteemed fellow citizen, Dr. Robert M. Porter, was adopted:

"Whereas it has pleased Almighty God to remove, by death, our fellow member, Robert M. Porter, and because of his devotion to the interests of the profession of medicine and his steady support of the American Medical Association,

"*Resolved*, That this Association has heard with unfeigned sorrow of his decease; and that they have lost a firm and intelligent supporter, and society a benefactor and friend."

The association then adjourned.

---

THURSDAY, May 7.

The association met according to adjournment, and the minutes of yesterday were read and approved.

The names of additional delegates were read to the association.

The secretary read a communication from C. G. Pease, of Wisconsin, which accompanied his report on Blending and Conversion of the types of fever. Referred to Committee on Voluntary Contributions.

Dr. Currey, from the Committee on Voluntary Contributions, made a partial report upon papers referred to that committee. Accepted.

Dr. J. B. Lindsley, from the Committee on Nominations, made a report of committees, etc., for the next meeting, place of meeting, &c. The report designates Washington city as the place for the next meeting. The first part of the report was adopted separately, and the latter part, after some discussion, was also adopted.

The following gentlemen were nominated in the report as secretaries for the ensuing year: Drs. R. C. Foster and A. J. Semmes, of Washington. The latter gentleman not being present, Dr. Brodie, retiring secretary, was requested to act as secretary, *pro. tem.*

Dr. C. K. Winston stated to the association that Mrs. Ex-President Polk would be pleased to receive calls from members of the association, from 4 to 6, P. M., to-day.

Dr. Pitcher offered a resolution providing the appointment of a committee of three, of which the president shall be chairman, to communicate with the Surgeon General of the Army, the Chief of the Medical Bureau of the Navy, and the Secretary of the Treasury of the United States, with a view to secure the concurrence of these departments of the Federal Government, so that the contributions to the medical topography, the vital statistics of the sanitary police of the nation, may be made tributary to the labors of this association. Adopted.

Dr. D. W. Yandell, of Kentucky, offered a resolution upon the subject of representation in the association, the gist of which was a provision requiring colleges, as a pre-requisite of representation, to require candidates for graduation honors to have attended at least two full courses of lectures, with an interval of not less than six months between them, one of which courses must have been in their institution. Dr. Yandell supported his resolution, and it was opposed by Dr. Buchanan, of Tennessee, who moved to lay it on the table.

Dr. Boling, of Georgia, offered a series of resolutions as a substitute, looking to the ruling of the subject of medical education out of the association entirely.

A discussion arose upon the resolutions somewhat protracted.

Dr. R. O. Currey offered a series of resolutions as a substitute, providing for a committee of five to report to the association a system of medical education.

After a prolonged discussion, the resolutions of Dr. Currey were adopted.

Dr. W. K. Bowling, from the Committee on Prize Essays, submitted a report awarding the premiums to H. F. Campbell, of Georgia, and Wm. A. Hammond, assistant surgeon U. S. Army, Fort Riley, Kansas.

Dr. Green, of Michigan, offered a resolution with a view to amend an article in the constitution Laid over.

The association accepted an invitation to visit the Medical College and the Military Department of the University of Nashville, in the afternoon.

The usual resolutions, complimentary to the citizens for their hospitality, to the reporters, the press and officers, were adopted, and the association adjourned *sine die*

